# Motivation to move fast, motivation to wait and see: The association of prevention and promotion focus with clinicians’ implementation of the JNC‐7 hypertension treatment guidelines

**DOI:** 10.1111/jch.14332

**Published:** 2021-08-10

**Authors:** Mechelle Sanders, Kevin Fiscella, Elaine Hill, Olugbenga Ogedegbe, Andrea Cassells, Jonathan N. Tobin, Stephen Williams, Peter Veazie

**Affiliations:** ^1^ Department of Family Medicine University of Rochester Rochester New York USA; ^2^ Department of Public Health Sciences University of Rochester Rochester New York USA; ^3^ Clinical Directors Network Inc Rockefeller Univ New York New York USA; ^4^ NYU Langone Health New York New York USA; ^5^ Clinical Directors Network Inc Albert Einstein College of Medicine New York New York USA

**Keywords:** clinical management of high blood pressure (HBP), hypertension, treatment and diagnosis/guidelines

## Abstract

Roughly half of the adults in the United States are diagnosed with hypertension (HTN). Unfortunately, less than one‐third have their condition under control. Clinicians generally have positive regard for the use of HTN guidelines to achieve HTN treatment goals; however, actual uptake remains low. Factors underpinning clinician variation in practice are poorly understood. To understand the relationship between clinicians’ personal motivation to complete goals and their uptake of the Joint National Commission's HTN guidelines. The authors used Regulatory Focus Theory (RFT, ie, prevention and promotion focus), an empirically supported motivational theory, as a guiding framework to examine the relationship. The authors hypothesized that clinicians with high prevention focus would report following guidelines more often and have shorter follow‐up visit intervals for patients with uncontrolled blood pressure. Clinicians (*n*  = 27) caring for adult patients diagnosed with HTN (*n* = 8605) in Federally Qualified Health Centers (*n* = 8). Clinicians’ prevention and promotion focus scores and the number of days between visits for their patients with uncontrolled systolic blood pressure (SBP) (≥ 140 mm Hg). Consistent with RFT, 60% of prevention focused clinicians reported they always followed the monthly visit guideline for the patients with uncontrolled blood pressure, compared with 38% of promotion focused clinicians (*p* = .254). The unadjusted probability of returning for a follow‐up visit within 30 days was greater among patients whose clinician was higher in prevention focus (*p* = .009), but there was no evidence at the 0.05 significance level in our adjusted model. These findings provide some limited evidence that RFT is a useful framework to understand clinician adherence to HTN treatment guidelines.

## 1 INTRODUCTION

Over 100 million adults in the United States (roughly 50%) are diagnosed with hypertension (HTN).^1^ Unfortunately, one‐third do not have their blood pressure controlled.^2^ This preventable condition costs about $131 billion per year.^3^ Studies have shown that patient non‐adherence contributes to suboptimal HTN control.^4^ Less appreciated is the role of clinician behavior in contributing to HTN control. Clinicians generally make two important decisions when treating patients with HTN. These include intensifying drug treatment when the blood pressure is not at goal and deciding how soon to schedule the patient to return for care.^5^

A Cochrane review of 72 randomized controlled trials compared various interventions for controlling blood pressure and found “overcoming physicians” hesitancy to prescribe complex medication regimens was the best strategy for decreasing average systolic and diastolic blood pressure by 8.0 mm Hg and 4.3 mm Hg,^6^ respectively. Clinician practice style drives visit frequency for chronic conditions such as HTN.^7^ Yet, little is known about what accounts for between clinician variations in these decisions.^8^, ^9^

We theorized that Regulatory Focus Theory (RFT) might be used to explain this clinical variation in adherence to HTN guidelines. RFT posits a person engages in two self‐regulating systems to achieve a goal, prevention, and promotion focus.^10^, ^11^ Prevention focus is associated with risk‐aversion and the need to meet obligations, whereas promotion focus is associated with risk‐taking and the need to explore options. A person high in prevention focus is motivated by meeting obligations, satisfying the expectations of other people, and maintaining safety.^10^ Adherence to a widely endorsed CPG is potentially the safer professional option in that it minimizes “looking bad” among peers and ostensibly prevents adverse outcomes among patients.^12^ Some clinicians might view CPG adherence as a professional responsibility.^13^

The relationship between RFT and HTN guideline adherence among clinicians has not been previously studied. The goal of this study was to understand the relationship between clinicians’ prevention and promotion focus and their uptake of the seventh Joint National Commission's (JNC‐7) recommendation of monthly follow‐up visits for patients with uncontrolled blood pressure.^14^, ^15^

## 2 METHODS

### 2.1 Sample

We used data from the Blood Pressure Visit Intensification Study (BPVisit) study, funded by the National Heart Lung and Blood Institute. A description of the intervention has been previously reported.^16^ The goal of the BPVisit study was to determine whether implementing a multimodal quality improvement intervention would result in clinicians’ uptake of the JNC‐7 guidelines that called for monthly visits for patients with uncontrolled blood pressure (≥140/90 mm Hg).^15^ We assessed 8605 unique, adult patients from BPVisit with uncontrolled blood pressure, across 27 clinicians from eight federally qualified health centers (FQHCs), which are members of Clinical Directors Network (CDN – www.CDNetwork.org), a primary care practice‐based research network, for their likelihood of returning to the FQHC within 30 days of having an uncontrolled systolic blood pressure (SBP) reading. The Institutional Review Boards at the University of Rochester and Clinical Directors Network approved the study.

### 2.2 Measurement of clinician prevention and promotion focus

We assessed prevention and promotion focus using Fellner and coworkers validated 10‐item Regulatory Focus Scale (RFS).^17^ The scale is composed of two subscales measuring promotion focus (eg, *“I prefer to work without instructions from others*”) and prevention focus (eg, “*Rules and regulations are helpful and necessary for me”)*. We summed the five items for each scale for a possible range of 5–35, with higher scores indicating higher proclivity for the given focus. Each clinician received a score for both prevention and promotion focus (continuous). We coded clinicians that scored high on the promotion scale but low on the prevention scale (classified by median split) as being predominantly promotion focused and vice versa.^18^

### 2.3 Assessment of clinician attitudes toward JNC‐7

We asked clinicians (physicians, nurse practitioners, and physician assistants) to complete a 20‐min survey to assess their knowledge about and attitudes toward adoption of the JNC‐7 guidelines prior to starting the BPVisit intervention. Items assessed their knowledge of the guideline for monthly follow‐up visits for patients with uncontrolled blood pressure, perceived effects of more frequent visits and perceived burden of monthly visits on patients.^16^ Clinicians also reported personal demographic information including their age, race and ethnicity, number of years in practice, Big‐5 personality traits,^19^ and any specialty training (ie, HIV).

### 2.4 Adherence to JNC follow‐up visit guidelines

We assigned patients with a SBP reading ≥ 140 mm Hg and returned to the FQHC within 1 month a score of “1” and those who did not return within a month a score of “0”, for all of their office visits during the study period.^14^ We included visits for all patients seen between January 4, 2014 and December 12, 2017 using data from their electronic health records (EHRs). Patient characteristics included age, sex, race, ethnicity, ICD‐9, 10 codes, language, diabetes, and chronic kidney disease (CKD) status.

### 2.5 Attributing a clinician to a patient

The clinician rendering care to a patient at a given visit was not necessarily the patient's primary care clinician. Therefore, some patients were linked to more than one clinician throughout the study period.

### 2.6 Data analyses

We used STATA statistical software (Version 12.0, Stata Corporation, and College Station, TX, USA) to conduct the analyses. We performed descriptive analyses on the clinician and patient characteristics. In our unadjusted logistic regression model, we examined the bivariate associations between the clinicians’ dominant prevention and promotion focus and whether a patient with uncontrolled blood pressure returned to the practice within a month. Next, we estimated an adjusted model using a generalized estimating equation with a Logit link function, a Binomial distribution, and exchangeable correlation matrix, which exhibited a low correlation of 0.03. We converted regression coefficients to odds ratios by taking the natural antilog of the logit coefficient. As a sensitivity analysis for the adjusted model, we used robust regression with an mm estimator and clustered standard errors.^20^, ^21^

We used one‐tailed significance tests to interpret the prevention and promotion focus coefficients, because our a priori hypotheses were directional, that is, prevention focus was positively associated with adherence to the clinical guidelines and promotion focus was negatively associated with adherence.^22^, ^23^ For the purpose of interpretation, we categorized results as providing weak evidence if the *p* value is between .1 and .05, moderate evidence if the *p*‐value is between .05 and .01, and strong evidence if the *p*‐value is less than .01.

The Big‐5 personality traits of the clinicians were included in the adjusted models to account for personality as a potential confounder. The clinician demographic variables included age, sex, number of years in practice, number of years at the FQHC, FQHC location and clinician training (physician vs physician assistant (PA), nurse practitioner (NP), or resident). Patient factors that were associated with pharmacologic management of HTN (age, non‐Black race, diabetes, and CKD) were included in the model.^14^ We controlled for mean SBP. We included a dummy variable for visit year to account for any lagged or temporal effects.

## 3 RESULTS

**FIGURE 1:**
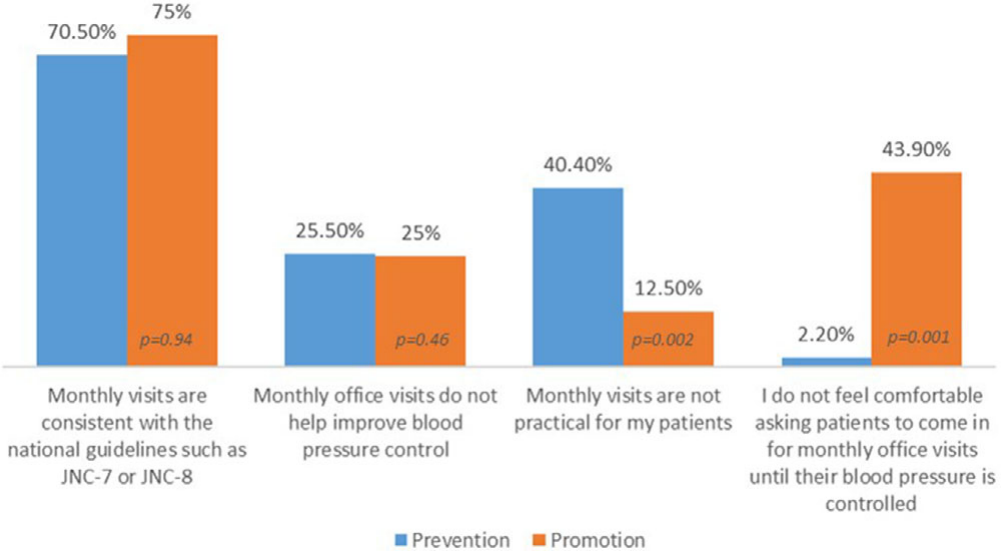
Percent of clinicians that responded they strongly agreed or agreed with statements related to knowledge around the JNC‐7 guideline for monthly visits, perceived burden of monthly visits on patients, and their comfort with requesting monthly visits

Table 1 displays the characteristics of the clinicians and patients. Most of the clinicians were physicians (60%), almost half (47.38%) had been in practice for 5 years or more, and 42% had worked at their respective FQHC for ≥ 5 years. The percent of patient visits per clinician ranged from 0.3% to 7.8% (mean = 3.3%, Std = 2.3%). The mean number of visits a patient had with the same clinician throughout the study period ranged from 28.5% to 100% (mean = 85.6%, Std 19.2%). The mean prevention score was 27.03 (range 18–34, median = 25) and the mean promotion score was 19.99 (range 15–27, median = 21). Fifty‐seven percent of the clinicians were classified as prevention dominant, 22% were promotion dominant, and 21% were not dominant on either scale. Forty‐seven percent of prevention dominant clinicians reported using the JNC‐7 guidelines at all visits with hypertensive patients, compared to 25% of promotion dominant clinicians. In addition, 60% of prevention dominant clinicians said they strongly agreed with the following statement “I generally schedule my patients whose blood pressure is not controlled to return in a month or less for their follow‐up visit”, compared to 38% of promotion dominant clinicians. However, the results did not reach statistical significance (*p* = .254). There were some statistically significant differences between the dominant focuses on perceived patient burden for monthly visits and self‐comfort with asking patients to come in for monthly visits. The majority of both promotion and prevention dominant clinicians knew monthly visits were consistent with the JNC guidelines (Figure 1).

**TABLE 1:** Characteristics of patients and clinicians

Patient characteristics (*n* = 8605)	
Age (mean) (Std)	60 years (12.33)
Race & ethnicity	
Black	55.75%
White	7.57%
Hispanic	13.91%
Female	78.35%
English speaking	85.92%
Diabetes	12.61%
Chronic kidney disease	0.44%
SBP (mean) (Std)	143.67 (17.15)
Between visit SBP variation (mean) (Std)	15.47 (13.40)
SBP controlled at visit (mean) (Std)	31.13% (24.30%)
No. office visits (mean) (Std)	10.16 (7.99)
No. of ICD9/10 codes documented at a visit (mean) (Std)	2.6 (6.33)
Number of days between each visit (mean) (Std)	116.89 (140.64)
Return visit within 30 days if uncontrolled SBP	27.49%
Clinician characteristics (*n* = 27)	
	mean (range)
Prevention focus score	27.04 (18–34)
Promotion focus score	19.99 (15–27)
Prevention dominant, above the median score (%)	57.05
Promotion dominant, above the median score (%)	21.27
Big‐5 personality traits	
Extraversion	4.15 (3.5–5.5)
Emotional stability	4.35 (3–5.5)
Openness	4.26 (3.5–6)
Agreeableness	4.53 (3.5–6)
Conscientiousness	6.30 (3.5–7)
Female clinician (%)	84
More than 5 years in practice (%)	47.38
More than 5 years at the FQHC (%)	42.34
MD (vs NP or PA) (%)	59.13

Roughly, 28% of the patients with uncontrolled SBP (≥140 mm Hg) returned for a follow‐up visit within 30 days. The patients had a mean age of 60 years old (range 20–99), were predominantly female (78%) and Black or African American (56%). The majority spoke English as their primary language (86%). Approximately 13% had an ICD‐9 or 10 code linked to diabetes and < 1% had a code linked to CKD. Patients had a mean SBP of 143.67 mm Hg and were seen at their FQHC approximately 10 times (range 3–64) over the course of the 44‐month study period.

### 3.1 Unadjusted probability of returning to the FQHC within 30 days if SBP is not at goal, by dominant focus

**FIGURE 2:**
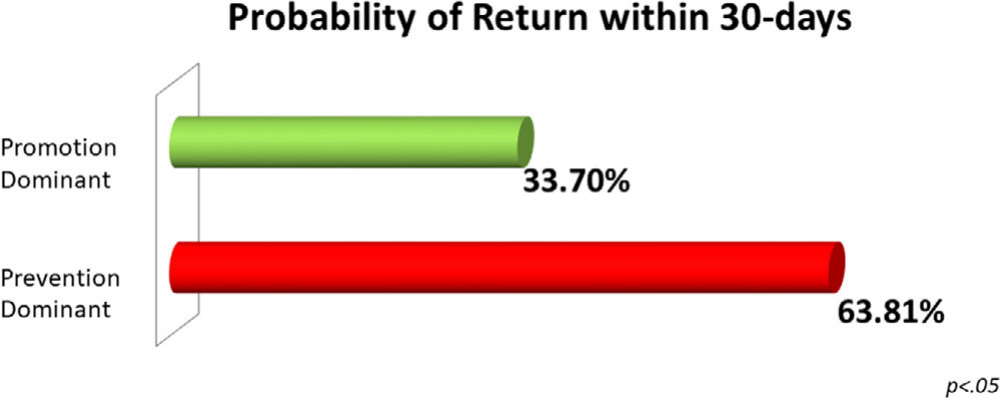
Percent of patients that returned for a visit to the FQHC within 30‐days of having an uncontrolled SBP reading, by their clinician's dominant regulatory focus. Approximately 34% of patients whose clinician was promotion dominant returned within 30‐days compared to 64% of patients whose clinician was prevention dominant

The relative risk of returning to the FQHC within 30‐days was 89% higher among patients whose clinician was prevention dominant, compared to clinicians that were promotion dominant (*p *= .009) (Figure 2).

### 3.2 Adjusted probability of returning to the FQHC within 30 days if SBP is not at goal, by prevention and promotion (continuous scores)

Table 2 presents the results of the explanatory variables from our adjusted generalized estimating analysis. There was weak evidence that higher prevention focus (1.04, one‐tailed *p* = .087) and higher promotion focus (0.954, one‐tailed *p *= .076) were associated with adherence to the 30‐day return visit guideline in the expected directions.

**TABLE 2:** Return to the FQHC within 30‐days

	Coef.	Std. Err	t	*p‐value*	[95% CI]	
Prevention	0.046	0.034	1.360	*.087	−0.020	0.111
Promotion	−0.048	0.033	−1.430	*.076	−0.113	0.017

*Indicates one‐tailed *p*‐value, adjusted for patient and clinician characteristics.

Table 3 presents the results of our sensitivity analysis. There was strong evidence that higher prevention scores were negatively associated with number of days between visits (‐0.247, one‐tailed *p* = .001) and moderate evidence that promotion focus was positively associated with number of days between visits (0.124, one‐tailed *p = *.015). The median number of days between visits for uncontrolled patients in 71 (approximately 2.3 months, and the mean was 117 (approximately 3.8 months).

**TABLE 3:** Number of days between uncontrolled BP visits

	Coef.	Robust Std. Err	t	*p‐value*	[95% CI]	
Prevention	−0.247	0.074	−3.35	*.001	−0.397	−0.097
Promotion	0.124	0.054	2.29	*.015	0.014	0.235

*Indicates one‐tailed *p*‐value, adjusted for patient and clinician characteristics.

## 4 DISCUSSION

Consistent with RTF, a majority (60%) of prevention dominant clinicians reported they always followed the monthly visit guideline for the patients with uncontrolled blood pressure. In contrast, fewer (38%) promotion dominant clinicians reported doing so.

In our unadjusted model, we found the relative risk of returning to the FQHC within a month was 89% greater among patients whose clinician was prevention dominant, compared to patients of clinicians were promotion dominant (*p = *.009). Recognizing that factors beyond the clinicians’ control influence whether a patient returns for a 30‐day visit, (eg, patient forgetting to schedule, scheduling constraints, and missed appointments) we also examined association between prevention focus and the number of days between visits. As hypothesized, higher prevention focus was related to the patient returning sooner. We could not confirm our hypothesis for promotion focus. Since only 28% of our overall sample returned for a visit within 30 days, it may be that monthly visits were not feasible for the FQHCs and the number of days between visits better reflects the clinician's ability to see patients for follow‐up.

To the extent that clinicians see the guidelines as obligations, these findings are consistent with, and explained by, RFT. For example, prevention focus is related to vigilant behavior and sensitivity to rules and regulations. Therefore, the watchful behavior associated with the monthly follow‐up visit guideline (more vigilance) would be expected to fit higher prevention focused individuals. On the other hand, promotion focus is related to risk‐tolerance.

### 4.1 Limitations

Our study has a few limitations that should be considered. First, our study sample of clinicians was modest which limited our power to detect differences. We had prevention and promotion focus data on 27 consented clinicians that completed the follow‐up surveys and their patients. Requiring follow‐up patient data eliminated less than 10% of the patient visits in our sample overall.

Second, the number of patients per clinician within FQHC varied. Some clinicians within an FQHC had upwards of 12% of our overall analytic sample, and some had as few as 0.4%. These differences in distribution mean less opportunity to detect variation for clinicians with fewer patients compared to those with more patients. Third, we know that HTN outcomes stem from a joint effort of the system, the clinician and the patient. The practice needs to have system in place to schedule patients for follow‐up visits. The clinician can make the recommendation, but the patient needs to show‐up for the appointment. The EHR data that we used in this study did not include whether a clinician suggested a follow‐up visit, we only had data on whether a patient returned for the appointment. Therefore, we are cautious as not to trivialize the importance of the patient's behavior in following through on completing the monthly visit. But, we argue it is a rare occurrence for the patient to request a follow‐up visit within a month, without a clinician's recommendation. This is especially true in the case of HTN where patients tend to be asymptomatic, and would not readily exhibit symptoms that would prompt them to return for further treatment on their own. In future studies, it will be important to include outcomes or address treatment decisions that are direct measures (rather than subsequent measures) of clinician behavior (such as ordering clinical laboratory) and explore reasons for patient delays in return that are beyond the clinician's control. Nonetheless, individual patient factors are not likely to impact physician chronic regulatory focus, which is a stable general disposition, and consequently will not confound the relationship between provider regulatory focus and patient behavior.

### 4.2 Study implications

Replication in a larger sample is needed to determine whether prevention focus affects clinicians’ adherence to HTN guidelines. A more direct test would involve assessing whether framing of CPG in either prevention or promotion language differentially affects clinicians’ attitude toward the CPG. For example, in their 2013 JAMA review paper, JNC‐8 tells the readers *”The following recommendations are based on the systematic evidence review…* *^14^* *”* Perhaps the fact that they are presented as recommendations rather than obligations makes them less important to clinicians. If the guidelines were presented as goals clinicians must achieve, rather than ought to strive toward, we may have seen greater uptake of use, and thus greater opportunity to test differences in the approach (prevention focus vs promotion focus) to achieve the goal. On the other hand, because the guidelines are written as recommendations, or ought's, they only cue prevention focus, as opposed to promotion focus. Qian and coworkers found most cardiologists were promotion focus dominant; whereas in our study, most primary care clinicians were prevention focus dominant.^24^ If these differences in distribution hold in other clinician specialties, guidelines could be drafted to appeal to the CRF type that dominates that specialty, writing guidelines using more prevention focused language for specialties where clinicians are more likely to be prevention focused, and vice versa. Further, we found that only 2% of clinicians higher in prevention focus were comfortable asking patients to come in for monthly visits until their blood pressure was controlled compared to 44% of those higher in promotion focus. Taken together, these findings seem to reinforce the basic premise of theory and the need to tailor interventions to meet the RF approach of the clinician.

## 5 CONCLUSIONS

Regulatory Focus Theory did show promise for understanding clinician adherence to HTN guidelines. These findings tell us that RFT could potentially be used to better understand variation in the uptake of other CPGs. Our work serves as a framework for future studies looking at the role of RFT in the clinical setting. Future studies should examine these associations in larger samples and determine whether how CPG are framed influence clinicians based on their regulatory focus.

## FUNDING

This trial was funded by the National Heart, Lung and Blood Institute (NHLBI) 1R18HL117801 (Fiscella, K; Tobin, JN)

## CONFLICT OF INTEREST

The authors report no conflict of interest.
